# Transcriptomics-Guided Personalized Prescription of Targeted Therapeutics for Metastatic ALK-Positive Lung Cancer Case Following Recurrence on ALK Inhibitors

**DOI:** 10.3389/fonc.2019.01026

**Published:** 2019-10-15

**Authors:** Elena Poddubskaya, Alexey Bondarenko, Alexander Boroda, Evgenia Zotova, Alex Glusker, Svetlana Sletina, Luidmila Makovskaia, Philipp Kopylov, Marina Sekacheva, Alexey Moisseev, Madina Baranova

**Affiliations:** ^1^Clinical Center Vitamed, Moscow, Russia; ^2^I.M. Sechenov First Moscow State Medical University (Sechenov University), Moscow, Russia; ^3^Faculty of Fundamental Medicine, Lomonosov Moscow State University, Moscow, Russia; ^4^FSBEI FPE Russian Medical Academy of Continuing Professional Education MOH, Moscow, Russia

**Keywords:** NSCLC, ALK, transcriptomics, personalized oncology, gene expression

## Abstract

Non-small cell lung carcinoma (NSCLC) is the major cause of cancer-associated mortality. Identification of rearrangements in anaplastic lymphoma kinase (*ALK*) gene is an effective instrument for more effective targeted therapy of NSCLC using ALK inhibitors dramatically raising progression-free survival in the *ALK-*mutated group of patients. However, the tumors frequently develop resistance to ALK inhibitors. We describe here a case of 48 y.o. male patient with *ALK*-positive NSCLC who was clinically managed for 6.5 years from the diagnosis. The tumor was surgically resected, but 8 months later multiple brain metastases were discovered. The patient started receiving platinum-based chemotherapy and then was enrolled in a clinical trial of second-generation ALK inhibitor ceritinib, which resulted in a 21 months stabilization. Following disease relapse, the patient was successfully managed for 33 months with different lines of chemo- and local ablative therapies. Chemotherapy regimens, including off-label combination of crizotinib + bevacizumab + docetaxel, were selected using the cancer transcriptome data-guided bioinformatical decision support system Oncobox. These therapies led to additional stabilization for 22 months. Survival of our patient after developing resistance to ALK inhibitor was longer for 16 months than previously reported average survival for such cases. This case shows that transcriptomic-guided sequential personalized prescription of targeted therapies can be effective in terms of survival and quality of life in *ALK*-mutated NSCLC.

## Background

Lung cancer is the most common type of cancer and the main factor of cancer-related mortality. According to the reports of World Health Organization and International Agency for Research on Cancer, in 2018 there were ~2.1 million new registered cases of lung cancer and ~1.8 million associated deaths (1). Non-small-cell lung carcinoma (NSCLC) is diagnosed in about 80–85% of all lung cancer cases. NSCLC response to standard chemotherapy (typically including treatment with platinum agents) is relatively poor with the median survival time of <1 year after diagnosis (2).

However, the identification of proper molecular biomarkers in NSCLC allowed to increase patient survival by specifically selecting targeted therapeutics. One of these biomarkers is rearrangement within the anaplastic lymphoma kinase gene *ALK* which is present in about 4–7% of all NSCLC cases (3, 4). Worldwide, ~40,000 new NSCLC cases with mutated *ALK* are detected annually. The current standards of care for patients with advanced *ALK*-mutated NSCLC include therapy with targeted ALK-specific therapeutic crizotinib and other selective ALK inhibitors (5). The median overall survival from the onset of treatment with crizotinib can reach 31 months (6). Although response rate on selective ALK inhibitors is high, there is a major problem of acquiring resistance to these therapeutics within 1–2 years from the onset of therapy (5).

In this report, we describe a case of *ALK*-positive NSCLC with brain metastases. The patient was under observation for 6.5 years and was treated by resection surgery, stereotactic radiosurgery, combination chemotherapies, and by several lines of targeted therapies. After the acquisition of resistance to crizotinib, two other targeted therapeutics were individually selected using a bioinformatic decision support system Oncobox based on the analysis of gene expression and activation of molecular pathways in the patient's tumor biosample (7, 8).

## Case Presentation

**Figure 1:**
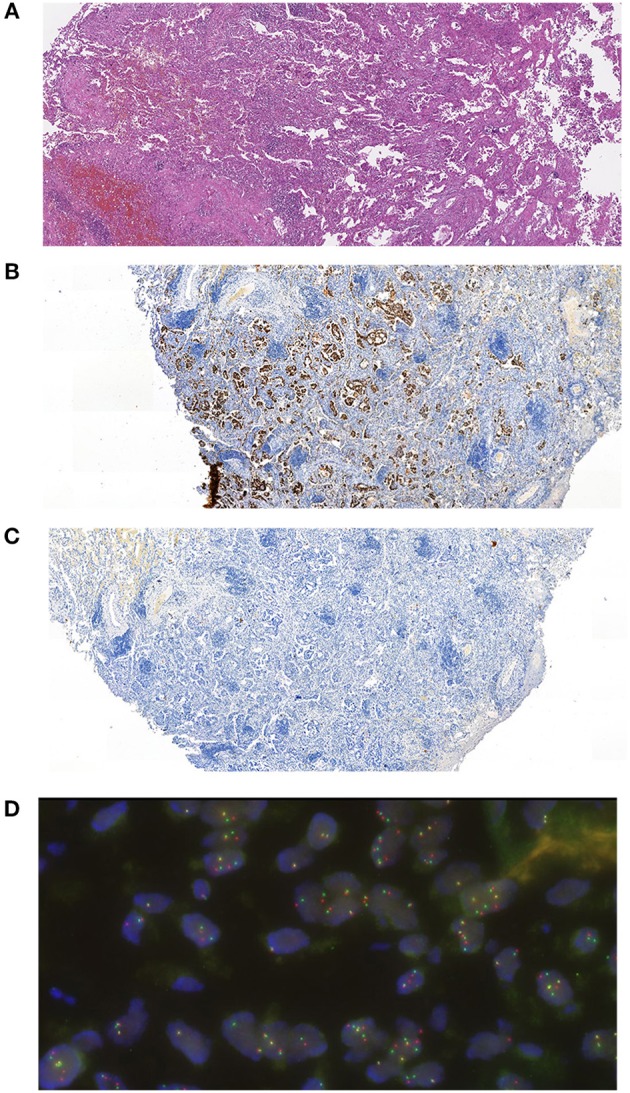
Histological evaluation of the patent's tumor. **(A)** Hematoxylin and eosin staining microphotograph. **(B)** Immunohistochemical staining for TTF-1 (SPT24). **(C)** Immunohistochemical staining for p40 (DeltaNp63). **(D)** FISH analysis for ALK-EML4 translocation.

The patient was 48 y.o. male diagnosed in January 2012 with *ALK* mutation-positive NSCLC, stage IIA, T2b N0 M0. The tumor has demonstrated positive immunostaining for TTF-1 (SPT24) and negative for p40 (DeltaNp63). ALK translocation was detected using FISH (Figure 1). The patient had a 10 pack-year smoking history but stopped smoking 3 years before the diagnosis. The patient underwent resection surgery (lower lobe of the right lung) and received 4 cycles of vinorelbine + cisplatin (25 mg/m^2^ IV on days 1, 8, 15, and 22 of a 28-day cycle with IV cisplatin 100 mg/m^2^ on day 1) as adjuvant therapy from February to May 2012.

Four months later (September 2012) the patient's condition worsened and multiple brain metastases were discovered (maximum size – 2.9 × 3.5 cm). In October-November 2012 the patient underwent whole brain radiation therapy (linear accelerator, a dose of 40 Gy in 2 Gy fractions) that resulted in a short-term stabilization with subsequent deterioration of the patient's condition.

In April 2013, following confirmation of *EML4-ALK* translocation, the patient was enrolled in the clinical trial NCT01283516 and was prescribed with a second-generation ALK inhibitor ceritinib (750 mg PO daily). Ceritinib therapy resulted in a reduction of brain metastases and the patient's performance status improved significantly. Five months later (September 2013) the patient was able to return to his professional occupation. In February 2015, after 21 progression-free months we observed an increase in the size of brain metastases and the patient was excluded from the NCT01283516 protocol according to exclusion criterion of neurologically unstable metastases.

**Figure 2:**
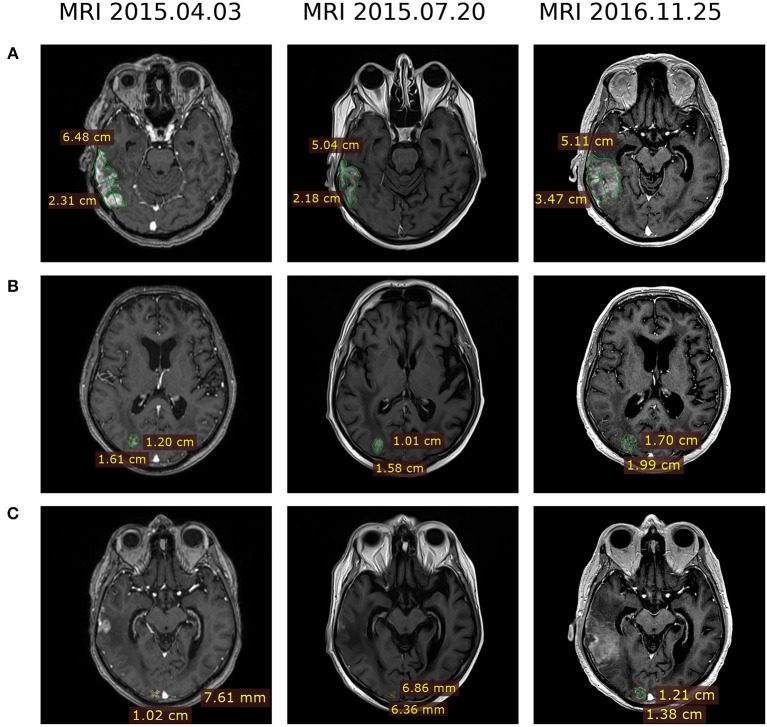
MRI evaluation of brain metastases dynamics at therapy lines 3 and 4 after development of the ceritinib resistance. Treatment regimens were pemetrexed + cisplatin (2015.04.03), topotecan followed by crizotinib (2015.07.20) and crizotinib + bevacizumab (2016.11.25). **(A)** Lesion 1, right temporoparietal region. **(B)** Lesion 2, right occipital lobe. **(C)** Lesion 3, right occipital lobe.

In March-June 2015, the patient received four cycles of pemetrexed + cisplatin therapy (500 mg/m^2^ IV on day 1 of each 21-day cycle), which resulted in a reduction of several lesions (MRI 2015.04.13, Table 1). After that four cycles of topotecan (2.3 mg/m^2^ PO days 1–5 of 21-day cycle) were prescribed followed by targeted therapy with first-generation anti-ALK drug crizotinib (250 mg PO twice a day). In July 2015, MRI evaluation revealed reduction of several metastases (Table 1, Figure 2).

**Table 1:** Brain lesions progression.

**Lesion**\**MRI date**	**2015.02.17**	**2015.04.13**	**2015.07.20**	**2015.09.10**	**2016.08.04**	**2016.01.24**	**2016.11.25**
Therapy	End of ceritinib	Pemetrexed + cisplatin	Topotecan, crizotinib	Crizotinib	Crizotinib, SRS + dexamethasone	Crizotinib	Crizotinib + bevacizumab
Lesion 1, right temporoparietal region	65 × 28 × 30 mm	65 × 23 × 25 mm	52 × 10 × 18 mm	61 × 29 × 29 mm	61 × 35 × 32 mm	61 × 34 × 30 mm	51 × 35 × 30 mm
Lesion 2, right occipital lobe.	8 × 9 mm	8 × 7 mm	6 × 7 mm	10 × 14 mm	10 × 14 mm	20 × 18 mm	20 × 17 mm
Lesion 3, right occipital lobe.	19 × 14 mm	19 × 14 mm	15 × 10 mm	25 × 17 mm	18 × 17 mm	14 × 12 mm	13 × 12 mm
Lesion 4, left parietal lobe, parasagittal.				5 × 5 mm	5 × 5 mm	5 × 5 mm	5 × 6 mm
Lesion 5, head of left caudate nucleus, paraventricular.	6 × 4 mm	6 × 5 mm	3 × 1 mm	6 × 6 mm	3 × 3 mm	Couldn't be visualized.	3 × 3 mm
Lesion 6, left temporal pole.				7 × 7 mm	16 × 11 mm	16 × 1.1 mm	16 × 11 mm
Lesion 7, left hippocampus.					4 × 4 mm	3 × 3 mm	3 × 3 mm
Lesion 8, left hippocampus.					4 × 4 mm	3 × 3 mm	3 × 3 mm
Sum of maximal diameters	99 mm	98 mm	77 mm	118 mm	125 mm	122 mm	115 mm
RECIST		Stabilization	Stabilization	Progression	Progression	Stabilization	Stabilization

*Changes in size of eight brain lesions from February 2015 till November 2016. Red color indicates increase of lesions in comparison to previous evaluation, green color indicates decrease*.

After 3 progression-free months, in September 2015, the patient's condition worsened (headaches, unstable walking). MRI examination showed an increase in size for all lesions previously identified (Table 1). We performed stereotactic radiosurgery for lesions in the right temporoparietal region (CyberKnife, a dose of 30 Gy in 6 Gy fractions) and other lesions (CyberKnife, a dose of 6 Gy). Dexamethasone (4 mg daily) was prescribed as adjuvant therapy.

To identify further possible options of chemo- and targeted therapy, we profiled gene expression in the patient's tumor biopsy using microarrays. Bioinformatical platform Oncobox was used to select potentially effective targeted drugs (9, 10). Following Oncobox report, bevacizumab was added to the treatment regimen since May 2016 (550 mg 7.5 mg/kg IV once in 3 weeks). This resulted in a decrease of several brain lesions (Table 1, MRI 2016.08.04, 2016.10.24, and 2016.11.25; Figure 2, 2016.11.25). In November 2016, the patient underwent another stereotactic radiosurgery for a lesion in the left parietal lobe (Novalis, a dose of 21.9 Gy).

In March 2017, after 10 progression-free months two lung metastases were discovered. Based on the Oncobox report, we added docetaxel to the treatment regimen (80 mg/m^2^ IV day 1 of 21-day cycle; 3 cycles). Due to significant adverse effects observed for the patient, the docetaxel dose was reduced to 60 mg/m^2^ for the next 3 cycles. In May 2017, CT examination showed reduction of lung lesions.

Following severe pneumonia in March 2018, the patient's condition significantly worsened. Examination in April indicated growth of all previously discovered lesions and the appearance of new metastases. Immunohistochemical testing revealed that ~98% of tumor cells were PD-L1-positive. The treatment scheme was changed in May 2018 and the patient received two infusions of monoclonal anti-PD-1 antibody pembrolizumab (200 mg IV day 1 of 21-day cycle). However, the patient's condition further deteriorated soon after immunotherapy administration. The patient died of brain edema in July 2018. The history of all treatments is summarized on Table 2.

**Table 2:** Outline of the patient treatment strategy.

**Date**	**Drug**	**Oncobox predicted drug efficiency score**	**Response**	**Progression free survival (months)**
**LINE 1**				
01–09.2012	Lung resection; vinorelbine + cisplatin	−7.65 (vinorelbine); NA (cisplatin)	No progression	7
10–11.2012	Whole brain radiation therapy		Progression	
**LINE 2**				
04.2013–02.2015	Ceritinib	0.6 (ceritinib)	Partial response	21
**LINE 3**				
03–06.2015	Pemetrexed + cisplatin	−0.5 (pemetrexed); NA (cisplatin)	Stabilization	3
06.2015–09.2015	Topotecan (4 cycles), crizotinib	0.55 (topotecan); 6.15 (crizotinib)	Stabilization	3
09.2015–05.2016	Stereotactic radiosurgery to multiple lesions; dexamethasone; crizotinib is continued	NA (dexamethasone); 6.15 (crizotinib)	Stabilization	8
**LINE 4**				
05.2016–03.2017	Crizotinib + bevacizumab Stereotactic radiosurgery to a single lesion	6.15 (crizotinib); 9.45 (bevacizumab)	Stabilization	10
**LINE 5**				
03.2017–03.2018	Crizotinib + bevacizumab + docetaxel	6.15 (crizotinib); 9.45 (bevacizumab); 0.45 (docetaxel)	Stabilization until severe pneumonia	12
**LINE 6**				
20.05.2018, 11.06.2018	Pembrolizumab	NA (pembrolizumab)	Not evaluable	No Data

## Materials and Methods

The patient provided informed written consent for gene expression analysis of his cancer biosample and for presentation of relevant clinical and molecular data in this paper. The tumor tissue sample used for gene expression analysis was obtained during lung resection in February 2012 and stored in the form of formalin-fixed paraffin-embedded (FFPE) tissue block at the room temperature. For RNA extraction, we used five 250-μm-thick consecutive sections of FFPE block with tumor cell content >80%. Gene expression profiling was performed using microarray platform CustomArray (USA) according to the Manufacturer's protocol, except for the addition of the dNTP mix containing biotinylated dUTP to the amplification reaction (final proportion of dTTP/biotin-dUTP was 5-to-1).

The expression profile of 3,682 human genes was measured and deposited in Gene Expression Omnibus repository with ID GSE133605. For gene expression normalization, four healthy lung tissue gene expression profiles from unrelated donors (GEO: GSM862609-GSM862612) were used as the reference (9). The signaling pathway activation analysis and prioritizing of targeted therapeutics were made using Oncobox bioinformatical platform, as previously described in Sanders et al. (10) and Poddubskaya et al. (11).

## Discussion

We report here the case of *ALK*-mutated NSCLC treated with six lines of therapy including several molecular-targeted drugs. Gene expression analysis complemented genetic *ALK* testing and was useful for selecting further treatment options.

The first line therapy was resection surgery and vinorelbine + cisplatin, which is the standard treatment for stage II NSCLC (12). The second line was monotherapy with ceritinib—the second-generation anti-ALK targeted drug currently recommended as the first-line therapy for *ALK*-mutated NSCLC (12) that was in the clinical trials back in 2013.

The patient developed resistance after 21 progression-free months. Acquired resistance is well documented for crizotinib, a first-generation anti-ALK target drug (13). Usually, ceritinib or other second-generation anti-ALK targeted therapeutics are used to overcome crizotinib resistance (14), and ceritinib resistance can potentially be reversed by using afatinib, another second-generation inhibitor of ALK (15). But back in 2015, second-generation ALK-inhibitors were at the different stages of clinical trials and the following treatment strategy was accepted.

First, pemetrexed + cisplatin polychemotherapy started because this regimen combination was reported to be effective and well-tolerated in NSCLC patients with brain metastases (16). Then the patient was treated with topotecan, a Topoisomerase I inhibitor that previously showed encouraging results in the NSCLC treatment (17). Finally, the patient was prescribed with crizotinib. To date discontinuation on ALK inhibitor therapy is not recommended because of possible disease flare (18, 19). Stereotactic radiosurgery was performed because local ablative therapy with continued administration of ALK inhibitors was effective according to the previous reports (20).

**Figure 3:**
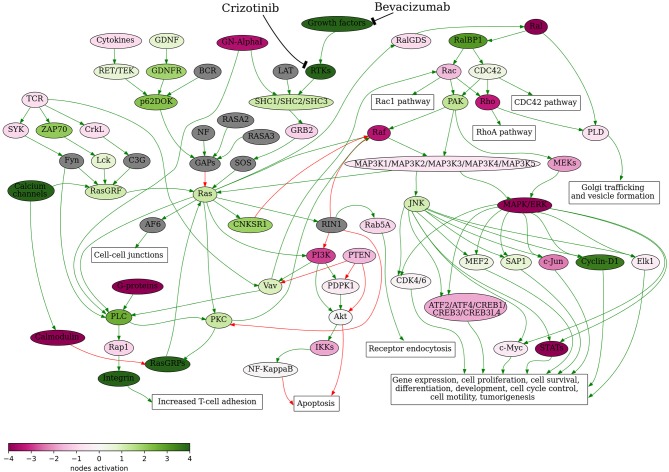
Ras signaling pathway shown as an interacting network. This pathway was hyperactivated in the patient's tumor tissue. Green arrows indicate activation, red arrows–inhibition. Transcript nodes are shown in ovals, interface molecular pathways and cellular effects–in rectangles. The color depth of transcript nodes reflects the extent of node activation (logarithms of the case-to-normal (CNR) expression rate for each node, in which “normal” is a geometric average between expression levels in normal tissue samples). Molecular targets of crizotinib and bevacizumab are indicated by black arrows. Visualization was implemented using Oncobox software.

It was recently demonstrated that genomic and transcriptomic profiling are potentially useful for improving therapy recommendations and patient outcomes (21). Identification of further therapeutic schemes including combinations crizotinib + bevacizumab and crizotinib + bevacizumab + docetaxel was based on the bioinformatic analysis of tumor gene expression profile. First, bevacizumab was added to the treatment scheme based on its positive simulated Drug Efficiency Score (Supplementary Table 1), which was calculated based on gene expression and activation level of molecular pathways in the patient's tumor using the Oncobox platform. It revealed that Ras signaling pathway was upregulated in the patient's tumor (Figure 3). The Ras pathway contains targets of both crizotinib and bevacizumab, so potentially the achieved clinical benefit of their combination may be linked with dual inhibition of this pathway.

Previously, activation of Raf-MEK-ERK signaling axis of Ras pathway was shown to be crucial for the *ALK* mutation-positive tumor cell survival and dual ALK-MEK inhibition was proposed as a new approach to battle tumor drug resistance (22). However, in the current tumor case the Raf-MEK-ERK axis was downregulated (Figure 3) and based on these data the dual ALK-MEK inhibition therapy would not be recommended.

Bevacizumab and other anti-vascular endothelial growth factor monoclonal antibodies were approved for the treatment of NSCLC (23). Recently, clinical investigation of crizotinib + bevacizumab combined therapy for advanced NSCLC reported a median progression-free survival of 13 months (24). In agreement with these results, in the case of our patient crizotinib + bevacizumab treatment resulted in 10 progression-free months.

When the patient progressed on crizotinib + bevacizumab therapy, docetaxel was added to the treatment regimen based on its positive simulated Drug Efficiency Score (Supplementary Table 1) and because of its different mechanism of action compared to the other therapeutics used. Docetaxel binds to microtubules, thereby interfering with cell proliferation and promoting cancer cell death. Docetaxel has been also approved for NSCLC (25) and bevacizumab + docetaxel polychemotherapy had a mean progression-free survival of 6 months for NSCLC in a published clinical investigation (26). However, to our knowledge, there are no previous reports on molecular-guided therapy with triple combination crizotinib + bevacizumab + docetaxel that resulted in 12 progression-free months in our case.

The next planned line of therapy was treatment with anti-PD-1 immunotherapeutic pembrolizumab since most of the patient's cancer cells were PD-1-positive. Unfortunately, severe pneumonia most likely accelerated further progression of the disease, and efficacy of the anti-PD-1 therapy couldn't be assessed due to the swift discontinuation of this treatment plan.

Overall, the patient lived for 78 months (6.5 years) after the diagnosis and 70 months after the discovery of brain metastases. The patient studies of ceritinib resistance development are only represented by several published clinical cases (27–29) and cannot be used to directly evaluate the effectiveness of our approach. However, there are far more literature data available for crizotinib. For male ALK mutation-positive patients treated with one or more lines of ALK inhibitors the median overall survival after stage IV diagnosis was found to be 48 months (30), while in the case of our patient the overall survival was 70 months. The patient's survival since the start of therapy with crizotinib (line 3) was 36 months which exceeds previously reported median values of 31 (6) and 16.6 months (31). Moreover, the median overall survival after progression on crizotinib was reported to be 25 months on next-generation ALK-inhibitors and only 6.4 months on the other therapies (31). However, our patient lived for 41 months after developing resistance to ceritinib and for 33 months after developing resistance to crizotinib, which is higher than both above estimates.

Therefore, this case suggests that the drug efficiency scoring based on gene expression profiling of the patient's tumor biopsy biomaterial could potentially complement the standard mutation analysis for the management of advanced cancer patients with NSCLC. In turn, the Oncobox platform can be potentially helpful for selecting effective treatment regimens also to the other types of solid tumors as previously shown for metastatic cholangiocarcinoma and ovarian cancer (9, 11).

## Data Availability Statement

The datasets generated for this study can be found in the https://www.ncbi.nlm.nih.gov/geo/query/acc.cgi?acc=GSE133605.

## Ethics Statement

The studies involving human participants were reviewed and approved by Institutional Review Board (IRB) at Clinical Center Vitamed, Moscow, Russia. The patients/participants provided their written informed consent to participate in this study. Written informed consent was obtained from the individual(s) for the publication of any potentially identifiable images or data included in this article.

## Author Contributions

EP, MB, SS, ABor, AG, and MS collected and interpreted patient data. EP, MB, and ABon were involved in clinical management. EZ and PK performed molecular analyses. LM and AM analyzed and interpreted the MRI data. AM, ABor, EZ, MS, PK, and EP wrote the paper.

### Conflict of Interest

The authors declare that the research was conducted in the absence of any commercial or financial relationships that could be construed as a potential conflict of interest.

## Supplementary Material

Supplementary Table 1 Oncobox balanced efficiency scores and pathway activation levels.
